# Does serum butyrylcholinesterase level determine the severity and mortality of COVID-19 pneumonia?: Prospective study

**DOI:** 10.3389/fmed.2022.940533

**Published:** 2022-07-25

**Authors:** Hilal Sipahioglu, Aliye Esmaoglu, Ayse Kiris, Zehra Bestepe Dursun, Sibel Kuzuguden, Mine Altinkaya Cavus, Cem Artan

**Affiliations:** ^1^Kayseri Education and Research Hospital, Kayseri, Turkey; ^2^Faculty of Medicine, Erciyes University, Kayseri, Turkey

**Keywords:** COVID-19, pneumonia, butyrylcholinesterase, marker, mortality

## Abstract

**Background:**

The WHO emphasized the importance of knowing the risk factors for the severity of the disease in the COVID-19 pandemic. Our aim in this study was to determine the relationship between serum Butyrylcholinesterase (BChE) level, which is rapidly affected by inflammation, and the severity of COVID-19 pneumonia and mortality.

**Methods:**

Patients diagnosed with COVID-19 pneumonia between March and May 2021 were included in the study. The patients were divided into two groups as severe and mild to moderate pneumonia according to the WHO's guidelines. Serum BChE levels were studied by ELISA method from the blood samples taken from the patients on the day of hospitalization. The severity of the disease and other factors affecting hospital mortality were also evaluated.

**Results:**

147 patients with COVID-19 pneumonia were included in this study. Of these patients, 58% had severe pneumonia and 42% had mild to moderate pneumonia. The BChE level was median 13 (IQR: 11.2–21.5)ng/ml in patients with severe COVID-19 pneumonia and median 20 (IQR: 10–35.7)ng/ml in patients with mild to moderate pneumonia (*p*: 0.001). Hospital with mortality rate was higher in patients with low BChE levels. However, statistically, BChE hasn't associated mortality in COVID-19 pneumonia [OR 1.002 (0.957–1.049) *p:* 0.490]. CRP, procalcitonin, lactate, and D-dimer levels were associated mortality in COVID-19 pneumonia.

**Conclusion:**

Being not statistically significant, the mortality rate was higher in patients with low BChE levels. BChE level is an important marker in determining the severity of COVID-19 pneumonia. Early prediction of the severity of COVID-19 pneumonia will enable early planning of the treatment process.

## Introduction

Coronavirus disease that emerged in 2019 is a global health emergency. It has been described as a pandemic by the World Health Organization. The World Health Organization emphasized that one of the most important questions to be addressed regarding the COVID-19 pandemic is to understand the risk factors for disease severity. Various prognostic and diagnostic biomarkers for COVID-19 have been proposed in the literature (1, 2).

BChE is an α-glycoprotein synthesized from the liver. Its level in the blood decreases in conditions such as liver damage, inflammation, and infection. BChE levels are strongly affected by inflammation, sensitively decreasing in the acute inflammatory phase and rising immediately when inflammation resolves. The cholinergic system plays an important role in maintaining and modulating an adequate immune response upon an inflammatory episode.

Cholinergic activity can modulate the magnitude of an appropriate immune response in an emerging inflammatory state (3–6). Therefore, immunity may be affected by the cholinergic system, which has an anti-inflammatory effect.

Levels of “positive” acute phase proteins such as C-reactive protein, amyloid A, and ferritin usually increase in patients with infection. Conversely, levels of “negative” acute phase proteins such as albumin, prealbumin, and transferrin decrease in response to infection and increase during the recovery period. Cholinesterase and lymphocytes act similarly to “negative” acute phase proteins in response to inflammation (7, 8).

Low lymphocyte ratio and high inflammatory markers affect the severity of COVID-19 pneumonia (9). In addition, CRP value is an independent risk factor affecting mortality (10).

Serum BChE level plays an important role in the inflammatory response, and it has been shown to be associated with prognosis in sepsis in a study by Peng et al. (11). There are not enough studies investigating the relationship between serum BChE level, mortality rate, and pneumonia severity in COVID-19 patients. In the study of Nakajima et al. (12), conducted for this purpose, the number of patients was small and the study was retrospective.

Our aim in this study was to determine the relationship between serum BChE level, which is rapidly affected by inflammation, and the severity of COVID-19 pneumonia and mortality.

## Materials and methods

Patients admitted to the intensive care unit and clinic with the diagnosis of COVID-19 pneumonia on March to May 2021 were evaluated. This study was approved by the local Ethical Committee of the university (No: 285).

Patients and patient relatives whose voluntary consent could not be obtained, patients with leukemia, liver cirrhosis, HIV infection, suspected and diagnosed with intoxication, and acute myocardial infarction were excluded from the study. The patients included in the study were divided into two groups as mild-moderate and severe COVID-19 pneumonia according to WHO interim guidance (13).

Demographic data and laboratory values of the patients were recorded on the first day of hospitalization. Blood samples were taken from the patients to measure the BChE level during hospitalization. Three ml blood samples were collected in tubes and the samples were centrifuged at 3000xg for 10 min. Then 1 ml of serum supernatant was removed and collected in eppendorf tube. Serum samples were kept frozen at −80°C. BChE serum protein concentrations were analyzed by using Elisa method (Cat. No E2519Hu).

## Statistical analysis

We used the statistical software IBM SPSS 24.0 for Mac (IBM Corp. Released 2016. IBM SPSS Statistics for MAC, Version 24.0., Armonk, NY, USA) for statistical analysis.

Categorical variables are presented as numbers and proportions, continuous variables as medians (interquartile range, IQR). Group differences in categorical and continuous variables were analyzed using chi-square and Kruskal–Wallis tests, respectively.

Comparisons of continuous variables between the groups were made using the Student's *t-*test or the Mann-Whitney U-test according to the conformity of the data to normal distribution. The groups were compared in respect to categorical variables using the Chi-square test or Fisher's exact test. To determine independent predictive mortality, forward step wise multivariate binary logistic regression analysis was applied by adding all the variables determined as *p* < 0.1 in the univariate analysis and the results were presented along with the odds ratio (OR) and confidence interval (CI).

## Results

One hundred and fourty seven patients who were admitted to the intensive care unit and clinic with the diagnosis of COVID-19 pneumonia between March and May, 2021, in our institution were included in the study. Mild and moderate pneumonia was detected in 62 (42%) patients and severe pneumonia in 85 (58%) patients. The median age (IQR) of the patients at diagnosis was 65 (52–78). Demographic and clinical characteristics of the patients are given in Table 1.

**Table 1:** Clinical characteristics of COVID-19 patients with mild—moderate and severe pneumonia.

**Variables**	**General (n: 147)**	**Mild-modarete pneumonia (n: 62)**	**Severe pneumonia (n: 85)**
Age, years	65 (52–78)	56 (46–67)	73 (63–83)
Male *n* (%)	70 (48)	25 (40)	45 (53)
Hypertension *n* (%)	68 (46)	23 (37)	45 (53)
Diabetes *n* (%)	46 (31)	17 (27)	29 (34)
Chronic obstructive pulmonary disease *n* (%)	34 (23)	12 (19)	22 (26)
Chronic kidney disease *n* (%)	19 (13)	3 (5)	16 (19)
Anything *n* (%)	44 (39)	28 (45)	16 (19)
Oxygen support *n* (%)	128 (87)	60 (97)	68 (80)
High flow therapy *n* (%)	26 (18)	2 (3)	24 (28)
Non invasive mechanical ventilation *n* (%)	26 (18)	2 (3)	24 (28)
Invasive mechanical ventilation *n* (%)	32 (22)	0 (0)	32 (38)
Steroid *n* (%)	52 (35)	26 (42)	26 (31)
Pulse steroid *n* (%)	13 (9)	7 (11)	6 (7)
Tocilizumab *n* (%)	20 (14)	6 (10)	14 (16)
Etanercept *n* (%)	7 (5)	2 (3)	5 (6)
APACHE II score	16 (11-22)	12 (8-14)	21 (16-27)
Hospital long stay d	13 (8-20)	11 (8-13)	18 (9-23)
Renal replacement therapy *n* (%)	25 (17)	0 (0)	25 (29)
Mortalite *n* (%)	32 (22)	0 (0)	32 (37)

*APACHE II, Acute Physiology and Chronic Health Examination*.

*In the patient group with severe pneumonia, the mean age of the patients was higher [56(46-67) vs 73(63-83), p: 0.001]. In patients with severe pneumonia, P/F(PO_2_/FiO_2_), BChE, albumin, and lymphocyte values were significantly lower, and CRP, procalcitonin, and white blood cell values were higher (Table 2)*.

**Table 2:** Risk factors affecting the severity of COVID-19 pneumonia.

**Variables**	**General (n: 147) Median (interquartile range)**	**Mild-modarete pneumonia (n: 62) Median (interquartile range)**	**Severe pneumonia (n: 85) Median (interquartile range)**	***P***
P/F ratio mmHg	164 (92–255)	278 (252–317)	110 (82–173)	<0.001
BChE ng/ml	16.23 (10.9–27.16)	20 (10–35.7)	13 (11.2–21.5)	0.001
Procalsitonin ng/ml	0.13 (0.06–0.48)	0.07 (0.04–0.09)	0.29 (0.11–0,97)	0.001
CRP mg/dl	52 (10.6–111.5)	12.95 (2.47–43.47)	98 (49.27–141.75)	<0.001
WBC /μL	9,500 (6,600–13,700)	7,550 (5,960–11,620)	11,700 (7,535–14,650)	0.001
Lymphocyte/μL	800 (560–1,310)	1,080 (727–1,580)	670 (490–1,195)	0.001
Platelet /μL	279,000 (165.000–312,000)	208,520 (155,000–286,957)	247,500 (191,000–333,250)	0.02
Creatinine mg/dl	0.8 (0.6–1.1)	0.7 (0.6–0.9)	0.9 (0.7–1.6)	<0.001
BUN mg/dl	15 (21–34)	17 (10–23)	29 (18–48)	<0.001
AST U/L	28 (20–43)	23.5 (18.7–30.5)	35 (21–52)	<0.001
ALT U/L	26 (16–45)	26.5 (17.5–43.5)	24 (13–45)	0.4
Albumin g/dl	3.3 (2.9–3.6)	3.6 (3.3–3.9)	3.1 (2.7–3.4)	<0.01
Ferritin ng/ml	373 (158–810)	213 (84–523)	573 (269–1056)	<0.001
Fibrinogen mg/dl	5,225 (4,047–6,732)	4,7153,430–6,037	5,605 (4,295–6,880)	0.005
INR	1.0 (0.9–1.1)	1 (0.90–1.05)	1.1 (1.0–1.3)	0.001
D-dimer ng/ml	707 (371–1,798)	411 (221–625)	1,208 (530–2,656)	<0.001
CK IU/L	53 (36.5–118)	41 (27–66)	82 (45–179)	<0.001
CKMB IU/L	28 (22–39)	23 (18.25–30.75)	33 (25–45)	<0.001
Troponin T ng/ml	11.3 (5.2–28.2)	6.3 (4–40,65)	23 (11–58.4)	<0.001

*ALT, alanine aminotransferase; AST, aspartate aminotransferase; BChE, Butyrylcholinesterase; BUN, blood urea nitrogen; CK, creatine kinase; CRP, C-reactive protein; INR, international normalized ratio; P/F, PaO_2_/FiO_2_ratio; WBC, white blood cell count*.

Ferritin, fibrinogen, D-dimer, creatine kinase (CK), CKMB, troponin, and lactate levels were significantly higher in the severe pneumonia patient group than in the mild-moderate pneumonia group.

The median APACHE II value of all patients was 16 (11–22). The APACHE II value was found to be significantly higher in the severe pneumonia group. The median number of days of hospitalization of the patients was 13 (8–20) days, and the number of days of hospitalization was significantly higher in the severe pneumonia group than in the mild-moderate pneumonia group (*p* < 0.001). Renal replacement therapy was required during treatment in 25 (17%) patients.

The hospital mortality of our patients was 32 (22%). All of our patients who died were in the severe pneumonia group, and BChE levels were found to be lower in these patients. However, when the risk factors for mortality of the patients were examined, BChE had significant effects in the univariate analysis [OR, (95% CI) 0.965(0.936–0.994), *p:* 0.026] while the effect on mortality was not significant in the multivariable analysis [OR, (95% CI), 1.002(0.957–1.049), *p:* 0.490]. Among the laboratory parameters, CRP, procalcitonin, and D-dimer values were independent risk factors affecting mortality significantly. The risk factors mortality are shown in Table 3.

**Table 3:** Blood parameters associated mortality in COVID-19 pneumonia.

**Variables**	**Univariable**	***p***	**Multivariable**	***p***
BChE	0.965 (0.936–0.994)	0.026	1.002 (0.957–1.049)	0.490
Procalsitonin	1.311 (1.002–1.715)	0.046	0.808 (0.659–0.909)	0.039
CRP	1.012 (1.007–1.018)	<0.001	1.014 (1.004–1.024)	0.019
WBC	1.118 (1.044–1.197)	0.002	1.040 (0.925–1.170)	0.165
Lymphocyte	1.098 (0.930–1.296)	0.400		
Platelet	0.997 (0,994–1.001)	0.081		
Creatinine	1.504 (1.119–2.021)	<0.001	0.511 (0.218–0.195)	0.971
BUN	1.060 (1.033–1.087)	0.007	1.063 (0.996–1.135)	0.238
Lactat	1.813 (1.216–2.704)	0.006	2.168 (1.056–4.449)	0.035
ALT	0.999 (0.994–1.004)	0.349		
Albumin	0.260 (0.117–0.577)	0.01	1.710 (0.518–5.643)	0.378
Ferritin	1.000 (1.000–1.010)	0.067		
Fibrinogen	1.000 (1,000–1.001)	0.049	1.000 (1.000–1.001)	0.982
INR	3.615 (1.172–11.149)	0.013	1.776 (0.318–9.908)	0.888
D-dimer	1.000 (1.000–1.001)	0.003	1.000 (1.000–1.001)	0.037
CK	1.000 (1.000–1.000)	0.867		
CKMB	1.003 (0.994–1.013)	0.765		
Troponin	1.000 (0.999–1.000)	0.649		

*Bold values are statistically significant*.

## Discussion

In this study, we showed that there is a significant relationship between the severity of COVID-19 pneumonia and the BChE level. BChE level was decreasing with inflammation, as were albumin and lymphocytes. According to the results of the multivariable analysis in our study, the most important factors affecting hospital mortality were CRP, procalcitonin, lactate, and D-dimer levels. In previous studies, D-dimer elevation was found to be associated with the severity and mortality of the disease and it was suggested that it should be used in the triage of patients (14–16).

There is a complex relationship between pro- and anti-inflammatory cytokines in response to inflammation. The release of pro- and anti-inflammatory cytokines from immune cells is partially regulated by the autonomic nervous system (17–19).

The role of afferent vagus nerve, efferent vagal and sympathetic nerve pathways in the regulation of inflammation has been shown in some studies (6, 20). After the vagal system is activated by inflammation, acetylcholine released from cholinergic axon terminals interacts with nicotinic α7 subunit receptors on immune cells, resulting in inhibition of pro-inflammatory cytokine release and restoration of immune homeostasis (6).

In our study, we also observed that the activation of the cholinergic system and the levels of cholinesterase, which degrades acetylcholine, were decreased in the patient group with more intense inflammation in COVID-19 disease, which progresses with intense inflammation.

In our study, the mean age of the patients in the severe pneumonia group was higher than the mild-moderate pneumonia group (*p:* 0.01). In the study conducted in a healthy geriatric population, no correlation was found between BChE levels and advanced age (21). Therefore, we can say that lower BChE levels in the severe pneumonia group are not related to age.

In previous studies, WBC, lymphocyte, procalcitonin, CRP, D-Dimer, CK, CKMB, and troponin levels in patients with severe pneumonia due to COVID-19 differed compared to non-serious patients (22, 23). In our study, we found that procalcitonin, CRP, WBC, lymphocyte, creatine, AST, albumin, ferritin, D-dimer, CK, CKMB, and troponin levels affected the severity of the disease.

Bahloul et al. (24) reported that cholinesterase levels were significantly reduced in septic shock patients and this result may be useful in the diagnosis of septic shock, but its prognostic value is weak. Peng et al. (11) reported that the mortality rate of patients with low cholinesterase levels was higher than those with normal cholinesterase levels in their study including 166 sepsis patients treated in emergency intensive care, and low cholinesterase level in sepsis patients was an independent risk factor for 30-day mortality. However, the study of Peng et al., was conducted retrospectively.

In our study, the BChE level was found to be effective in showing the severity of COVID-19 pneumonia. In this study, independent risk factors affecting hospital mortality were determined as procalcitonin, CRP, lactate, and D-Dimer levels.

The limitations of our study were that it was conducted in a single center and the number of patients was relatively limited.

## Conclusion

Although serum BChE level is not statistically significant in predicting mortality in COVID-19 pneumonia, it is an important marker in determining the severity of COVID-19 pneumonia. Early prediction of the severity of COVID-19 pneumonia will enable early planning of the treatment process. We think that further multicenter studies are needed on this subject. Early prediction of pneumonia severity will be important in-patient treatment, patient triage, and timely admission of the patients to intensive care during the pandemic process.

## Data availability statement

The original contributions presented in the study are included in the article/supplementary materials, further inquiries can be directed to the corresponding author/s.

## Ethics statement

The studies involving human participants were reviewed and approved by Kayseri City Training and Research Hospital of Ethics Committee No: 285. The patients/participants provided their written informed consent to participate in this study.

## Author contributions

Conceptualization: HS. Formal analysis: HS and AK. Visualization and writing—original draft preparation: HS and AE. Writing—reviewing and editing, data curation, methodology, and validation: All authors. All authors contributed to the article and approved the submitted version.

## Conflict of interest

The authors declare that the research was conducted in the absence of any commercial or financial relationships that could be construed as a potential conflict of interest.

## Publisher's note

All claims expressed in this article are solely those of the authors and do not necessarily represent those of their affiliated organizations, or those of the publisher, the editors and the reviewers. Any product that may be evaluated in this article, or claim that may be made by its manufacturer, is not guaranteed or endorsed by the publisher.
